# Consumer Profile and Product Knowledge Affect the Usefulness of a Quality Label as a Tool to Differentiate a Product: A Chilean Survey

**DOI:** 10.3390/foods10071482

**Published:** 2021-06-25

**Authors:** Begoña Panea, Ignacio Subiabre, Andrea Haudorf, Rodrigo Morales

**Affiliations:** 1 Unidad de Producción y Sanidad Animal, Centro de Investigación y Tecnología Agroalimentaria de Aragón, Avda, Montañana 930, 50059 Zaragoza, Spain; 2 Instituto Agroalimentario de Aragón–IA2, CITA-Universidad de Zaragoza, 50013 Zaragoza, Spain; 3 Instituto de Investigaciones Agropecuarias, INIA Remehue, Ruta 5 Sur Km. 8, Osorno 5290000, Chile; ignacio.subiabre@inia.cl (I.S.); a.haudorf@gmail.com (A.H.); rmorales@inia.cl (R.M.)

**Keywords:** meat, consumer, quality label, perception, online survey

## Abstract

Quality labels are useful tools to differentiate food products, but only if consumers recognise them and associate them with specific characteristics. An online survey was conducted to investigate whether Chilean consumers knew about Novillo de Osorno, for which a quality label is being developed. The survey was divided into five blocks: lifestyles, meat consumption and purchase habits, meat choice behaviour, knowledge about Novillo de Osorno, and consumers’ socio-demographic information. The place of residence and consumer gender, age, or income were important cues in defining consumers’ lifestyles, meat consumption and purchase habits. Respondents could be grouped into three main groups: 1. Younger people: urban with medium-high incomes, which search only for pleasure; 2. Foodies uninvolved and Females uninvolved: females with the highest income level that chose food for nutritional reasons; and 3. Traditional people: men older than 55 with low incomes, living in the Northern areas and interested in taste and in the meat’s origin. Nearly 60% of respondents had never heard about Novillo de Osorno. Hence, the supply chain has an opportunity to extend the market. Since price and confidence in the origin are important cues, they must be considered in the design of promotion strategies.

## 1. Introduction

Quality labels provide information regarding traditional food in localities and communities and allow the maintenance of knowledge, traditions, and diversity [1]. Quality labels are a recognized tool to differentiate the quality of meat and meat products in a global market. Although their development in Chile is new and incipient, differentiation by origin has evolved from being ignored to having increasing importance and internationalization in the last years. The implementation in 2005 of the national Law N° 19-996 [2] introduced some modifications of intellectual property, being the first law concerning specifically the quality labels. Then, Chile began to give to some products a recognition based on the reputation fundamentally due to their geographical origin, also including natural and human factors. For example, there is a label named “Certificación Calidad Aysén Patagonia Chile” for the products manufactured with raw material from the area. Currently, there are in Chile two certified meat products: “Cordero Chilote” (lamb) and “Prosciutto de Capitán Pastene” (cured ham) and some studies are being carried out to obtain a new quality label for the beef “Novillo de Osorno” [3,4], which includes the name of the production area, placed in the southern region of the country.

The Southern Chilean regions have the environmental conditions to produce grass-based meat, which is traditionally considered by the consumers as more natural meat, as well as a healthier meat [5,6]. Nevertheless, in the supermarkets, the most commonly sold meat comes from Argentina, Brazil, Uruguay, and Paraguay, where the animals are commonly reared, housed, and given a feed based on cereal. To inverse this situation could be implemented by the creation of quality labels for national meat.

Nevertheless, the acceptance of new food products is mediated by expectations and previous experiences, as well as the factor of belief, or credence quality [7,8]. Most studied credence attributes of meat include origin, animal welfare, production system/feeding, health/nutrition, and environmental issues [9]; nonetheless, this information is not always available during purchasing and even consumption [10,11]. Hence, the consumers search for information through the mass media, advertisements, word of mouth, etc. [12,13,14], but the increase in the urban population in the last decades, distancing people from the productive environment, has generated insecurities regarding the quality of the product by the consumers [15]. In addition, consumers are increasingly more attentive to the values and identity of the meat brand and they hope to align themselves with their values [16].

These changes in food consumption patterns are due not only to socioeconomic and cultural trends but also to the specific lifestyles of consumer groups. Lifestyle is defined by sociodemographic variables (age, gender, income…), beliefs, attitudes, habits, health and environmental concerns, and knowledge and involvement with the product, as well as consumer shelf image. Grunert et al. [17] established as a methodological framework the food-related lifestyle (FRL) as a mediator between consumer values and their behaviour. An FRL is composed of five interrelated elements: how to make purchases, quality aspects for the evaluation of food products, cooking methods, consumption situations, and the reasons for purchase. The FRL theory and its posterior modifications and variations has shown that consumer behaviour and purchase decisions are mediated by their lifestyle, and it has been widely used in food science to study consumer perception [18,19,20,21,22,23,24].

This association, between health and personal and family well-being and food quality, could enhance the opportunities to capture the value of the environmental and social sustainability attributes through the market [25], and the use of quality labels could be a tool to recuperate the sureness about meat quality and to promote the consumption of national meat.

Therefore, the aim of the current study was to investigate, by means of an online survey, the Chilean consumer knowledge about Novillo de Osorno meat and whether it is influenced by consumers’ lifestyles and meat consumption habits.

Given the exposed conceptual framework, this research is based on three aims:

- Aim 1. To study if the region in where people live affects lifestyle, meat consumption habits, and the knowledge of Novillo de Osorno.
- Aim 2. To study if the socio-demographic variables (number of adults and minors at home, consumer gender, age, and income) affect lifestyle, meat consumption habits, and the knowledge of Novillo de Osorno.
- Aim 3. To study if consumers can be clustered based on their lifestyle and meat consumption habits and described in terms of socio-demographic variables.

Aims 1 and 2 will help us decide which variables are important when constructing aim 3.

## 2. Materials and Methods

### 2.1. Questionnaire

An online survey was carried out using the Google survey tool. The questionnaire was validated by researchers uninvolved in the study and adapted until the final version was approved.

The survey is based on an FRL model adapted to Chilean idiosyncrasies. The FRL consists of 69 Likert-type attitudinal items, rated on a 7-point scale, and collapsed into five different domains of food-related lifestyle factors: ways of shopping, cooking methods, quality aspects, consumption situations, and purchasing motives. Nevertheless, a 7-point scale is not easy to use for many people, mainly in an online unsupervised survey, in which respondents have the help of the interviewer. Therefore, our survey was also divided into five blocks but following the methodology of Bernués, et al. [1]. The closed-response questionnaire comprised different types of scales instead of the 7-point scale defined in FRL theory. The first block investigated lifestyles and included 21 true and false questions, concerning food consumption and purchase behaviour. The second block comprised meat consumption and purchase habits and asked about the kind of meat usually consumed, the place of buying, the origin of the purchased meat, and the reasons to buy imported meat, with multiple options and only one possible choice. The third block was a conjoint analysis for meat choice behaviour, showing two series of 4 pictures each, with indication of the meat joint, commercial category, rearing system, and price, and consumers were asked to choose one of the scenarios for each series of pictures. The fourth block aimed to investigate the consumers’ knowledge about Novillo de Osorno and included two questions without information, followed by an explanation of what Novillo de Osorno is and two posterior questions about willingness to purchase Novillo de Osorno. This block also had multiple answers with a unique choice option. Finally, the fifth block contained some socio-demographic information (place of residence, age, gender, number of children/adults at home, and income). Used survey is in Table A1.

### 2.2. Recruitment

The questionnaire was distributed using social media websites, university mailing lists, and survey mailing lists. A divulgation message was included that asked receivers to further broadcast the web link to other persons in their respective electronic mailing lists. For those receivers willing to participate in the survey, the survey welcome page was immediately accessible after they clicked on the web link. The questionnaire was anonymous to guarantee a higher level of participation and honesty. Personal data, such as identification or electronic mail address, were not required, and there was no financial compensation. Participants were clearly informed of the aim of the study and gave implicit consent for the use of their supplied information in the research.

### 2.3. Statistical Analysis

The received answers were filtered according to two requirements: to be over 18 years old and not to be vegetarian or vegan, obtaining a total of 615 valid answers. Consumer age was grouped into three categories (18–34 years old, 35–50 years old, 51–83 years old), accordingly with data from the National Institute of Statistics (www.ine.cl, accessed on 19 August 2020). Residence city was recorded as the region of belonging, accordingly with the country’s administrative divisions.

Data were analysed using XLStat v 2014.3.05 (Addinsoft, Barcelona, Spain), The frequencies of each variable were calculated. To interpret the patterns of association between the studied variables, a chi-square test was used and the corrected standardised residual between the observed and expected cases within each cell greater than |1.96| was considered. Two Kruskal–Wallis tests with a 0.05 significant level were carried out to study the different hypotheses, that is the influence of the region where people live or the socio-demographic variables (number of children and adults at home, consumer gender, age, and income) on consumption lifestyle, on consumption and purchase habits, and on Novillo de Osorno knowledge. To cluster the consumers, three multiple factorial analyses (MFA) with lifestyle and meat consumption habits were performed; the first one, considering all data, the second one considering data from people living in the Metropolitana de Santiago region, and the third one, considering people living in other places, different from the Metropolitana de Santiago region. In each of the clustering processes, variables whose sum of cosines in the two first dimensions was higher than 0.5 were included in the subsequent agglomerative hierarchical cluster. Then, a Kruskal–Wallis test with a 0.05 significance level and the frequencies of the answers was performed to investigate the differences between clusters regarding lifestyle, meat consumption and purchase habits, meat choice behaviour, and socio-demographic variables.

## 3. Results and Discussion

### 3.1. Exploring Global Data

Global frequencies for each of the answers as well for some of the socio-demographic variables are in Table A1. The sample was equally distributed by gender (49.8% men) and the distribution by age fit with the country distribution; the percentages for the three age groups (18–34, 35–50, 51–83) were 33.3, 36.3, and 30.4%, respectively, in the sample, and 26.5, 21.6, and 26%, respectively, in the country. 

Concerning the place of residence, comparisons between sample and country are in Table 1. Regions of Biobio, Los Lagos and Metropolitana de Santiago were slightly overrepresented in the current sample but in general, the sample was representative of the country distribution. Since the Metropolitana de Santiago region represents almost half of the country, we proposed the hypothesis that people living in Metropolitana region have different behaviour from the others, which will be discussed in the following subheading.

Regarding the structure of the families (number of adults and minors at home, Table A1), 57.9% of respondents declared no minors at home, 24.9% had one minor, 11.5% had two, and 4.9% had three. Only 0.9% declared to have more than three minors at home. For adults, the most frequent number was two (43,4%), followed by three adults (20.3%), four adults (16.1%), and a single adult (12.0%). The remaining 7.8% registered five or more adults. Therefore, most of our respondents seems to be families composed of a couple, and their descendants, with or without their older parents. The importance of the number of minors will be investigated in the aim 2.

The sample distribution by income is in Table A1. The average monthly income in Chile was CLP (Chilean pesos) 573,964 in 2018, whereas the median income reached CLP 400,000 per month (National Statistics Institute). Nevertheless, the current sample had 32.5% of respondents with incomes higher than CLP 1.5 million, that is, over USD 1900 (CLP 1000 is approximately USD 1.26) and the median of the current sample was CLP 750,000–1 million. This overrepresentation of higher-income homes could affect the consumer behaviour and will be investigated in aim 3, laterally with gender and age affects.

The percentage of valid answers for lifestyle questions, for meat consumption habits, and for meat choice behaviour are also in Table A1. From lifestyles, Chilean consumers could be defined as people who love to purchase food, to cook and to try new recipes, and who search for pleasure and taste when eating. On the other hand, they give importance to meetings with family and friends, prefer traditional recipes to new ones, and are concerned about the Chilean identity of food.

Most people ate all kind of meats (74.1%) and bought it at the supermarket on trays (44.8%) or in a traditional butchery (28.7%). They bought national or imported meat, depending on the offer, and the main reasons to buy imported meat are the price (33.7%) and that imported meat is easier to find (32.2%). When buying meat, they preferred grass-fed than grain-fed, even if the grass-fed option was more expensive than the grain-fed option.

Of the respondents, 56.7% had never heard about Novillo de Osorno and when they knew it, they had no clear idea of what Novillo de Osorno was, since only 36.1% answered that it was a kind of meat, whereas 26.4% believed that it was dairy cattle, and 14.5% recognised not knowing exactly what it was. Once informed about what Novillo de Osorno was, only 14.8% of respondents considered that the meat they buy is Novillo de Osorno, although another 19.2% declared buying it sometimes. Most people would buy Novillo de Osorno if they found it in the market, at least sometimes, depending on price and piece (25.5%), the origin being the attribute most important in choosing it (32.4%).

Several practical implications can be drawn from the general data. Firstly, more than 74% of people stated to eat all kind of meat and so they could be considered as potential Novillo de Osorno customers, but 57% of them had never heard about Novillo de Osorno, which in turn, implies an opportunity to extend the market. In addition, nearly 26% of the people that declared to know Novillo de Osorno were wrong about what it was, which implies the necessity of a marketing strategy to inform consumers of what Novillo de Osorno really is and, consequently, an opportunity for market growing.

On the other hand, considering that 57% of people buy their meat at the supermarket or in a traditional butchery, promotion campaigns should be implemented in these areas to promote the consumption of Novillo de Osorno. Nevertheless, nearly half of people declared not to read the labels and, therefore, several complementary strategies must be implemented. In any case, price is an important key for most people, and it must be considered in the design of these promotion strategies. In the same way, the main reason to buy Novillo de Osorno is the confidence in the origin, which is a strength that must be enhanced in these marketing strategies.

Finally, an important finding of the study was that 32% of people bought both indistinctly national and imported meat. There are two main reasons to buy imported meat: it is cheaper than national products, or it is easier to find than Chilean meat. In fact, between 2007 and 2017, Chilean beef production decreased at an average annual rate of 1.7%, and, in 2017, 60% of the beef consumed in Chile was imported, suggesting a lack of competitiveness in the industry [26]. Since people buy imported meat because it is easier to find than national meat, the Chilean supply chain has an opportunity to increase the internal consumption of Chilean meats. In addition, 19% of respondents thought that foreign meat has better quality than Chilean meat, which implies that the suppliers should make an effort to transmit to the population the actual quality of Chilean meat. Nevertheless, since price is important for a third of consumers, two plausible methods are conceivable: 1. some political decisions could be made to protect national products and to allow farmers to offer better prices, or 2. a marketing strategy based on quality attributes must be developed to explain to consumers why they should choose Chilean meat, even if it is more expensive than foreign meat.

### 3.2. Objective 1. The Region Where People Lived Influenced the Lifestyle, the Meat Consumption/Purchase Habits, and the Meat Choice Behaviour

Some studies reported that place of residence and socio-economic context influenced choice and patterns of behaviour [27]. A Chilean study demonstrated that the region of residence modified the importance attributed to the different cues; for example, consumers from La Araucanía (south) showed higher ethnocentricity and greater preference for Chilean meat than people from the Metropolitana region, whereas consumers from the Metropolitana area were more concerned with price than consumers from other regions [28]. Then, three different tests were carried out: considering all the regions (*n* = 15), comparing the Metropolitana de Santiago region vs. the other regions, and among the regions but excluding the Metropolitana de Santiago region. Almost all considered variables were affected by the region in where people lived, independently of whether Metropolitana de Santiago was considered in the model or not. Nevertheless, there was not a clear pattern, and we could not see a region that was clearly different from the rest in all lifestyle variables, which could be partially explained on the basis of the geographical complexity of the country [29]. Then, we decided to complete three different clustering processes (Objective 3). The frequencies of answers for each item in each cluster are shown in Table A2, Table A3 and Table A4.

### 3.3. Objective 2. The Socio-Demographic Variables (Number of Children/Adults at Home and Consumer Gender, Age, and Income) Influenced Lifestyle, Meat Consumption/Purchase Habits, and Meat Choice Behaviour

The effect of the family structure (number of minors and number of adults) is in Table 2. The effect of number of children was less noticeable than the effect of number of adults. Number of children at home affected the importance give to mealtime as well to the place of food purchase. Families without minors at home considered mealtime in the family less important than expected (78% instead of the expected 81%). On the other hand, families with two minors at home bought the meat preferably in a traditional butchery (48% instead of the expected 29%) and bought less than expected in the slaughterhouse (26% vs. the expected 45%).

Regarding adults, families with two adults at home ate at work less than expected (66% instead of 70.8%), whereas the opposite occurred in families with five adults (91% instead of the expected 72%). It must be noted that 41% of families with two adults declared incomes higher than CLP 1.5 million and only 32% declared incomes lower than the country median (CLP 500,000–750,000), whereas 47% of families with five adults are in this medium spectrum of incomes. 

Households with a single adult did not spend less time on cooking (49% vs. the expected 30%) whereas homes with two adults did (25 vs. 30%), which could be due to differences in respondent gender, since in unipersonal homes, the percentages of men are higher than the percentages of women (55 and 45%, respectively), whereas the opposite happened in homes with two adults (49 and 51%, respectively). Additionally, people with higher incomes (more than CLP 1.5 million) spent more time on cooking (12% more than expected), which is consistent with the observation that they eat mainly at home.

From current data, we can conclude that the number of minors as well as the structure of the family has little influence on purchase and consumption habits. Therefore, it is not necessary to take them into account in clustering models. In addition, since the influence of the family structure, defined in terms of number of minors and adults at home, seems to depend on the consumer gender, age, and income level, only consumer gender, age, and income were considered in the clustering.

Gender affected eight of the items. Although both men and women liked to buy food and to cook, the percentages were higher for women then for men. In addition, women chose food for nutritional reasons more frequently than men. Several studies showed that women are more concerned about the link between food and health or social image, which could explain these differences [30,31,32]. Regarding age group, as expected, young people were more likely to try new products and recipes, eat out for pleasure, and cook recipes from other countries than older people. Accordingly, young people have less interest in products with Chilean identity, they do not have fixed habits, and they give little importance to mealtime. These results agree with a study carried out in the Metropolitana de Santiago area that concluded that a lifestyle where eating is related to enjoyment of food is associated with a person’s higher socioeconomic level and lower age [27]. On the other hand, older consumers declare not to use advertising and not to read labels, maybe because older people have more experience buying food [33].

Lifestyle is also affected by income level. People with higher incomes declare to trust in advertising, read the labels, choose food for nutritional reasons, like new products and recipes, eat out for pleasure, and spend time cooking, but they are not concerned about taste, price, Chilean identity, mealtime, or traditional recipes. Just the opposite occurs with people with incomes at the average of the country. On their own, lower-income people ignore advertising and do not like trying new products or recipes. Some studies showed that income level influenced the buying habits of meat [34] and that people with low or intermediate levels of education prefer to buy only known products [35].

### 3.4. Objective 3. Consumers Can Be Clustered Based on Their Lifestyle and Meat Consumption Habits and Described in Terms of Socio-Demographic Variables

Three multiple factor analyses were carried out, considering all data, only data from the Metropolitana de Santiago region, or data from regions different from the Metropolitana de Santiago region. For the three MFA, around 12% of the variability was explained by the two first factors of each of them. Only some lifestyle variables were discriminant, whereas meat purchase consumption and habits were not. Liking for buying food, label information, liking for cooking, time spent on cooking, importance of taste, nutritional value, and liking for foreign or new recipes had higher contributions to the three MFA. With these variables, we constructed the three corresponding clusters.

#### 3.4.1. Clusters with All Data

Three classes were obtained from the hierarchical cluster. The clustering separated clearly the people by function of the residence region (north, centre, south) and income (low, medium, and high, respectively). Frequencies for the answers in each cluster are in Table A2.

The first group contained 80 respondents and included people aged < 35 years, with incomes within the country mean (CLP 750,000–million) and living almost exclusively in the Metropolitana de Santiago region. We can name them Young urban. Regarding their lifestyle, they like neither to buy food nor cooking, and they spend a low amount of time on it. They do not use purchase lists, they trust in advertisement, they consider taste to be more important than health, and they love to eat out of the home for pleasure, although they also did so because of work. Family meals are unimportant for them, they do not have fixed habits, and they do not like foreign recipes. They eat mainly white meat and buy it in traditional butcheries, the origin of the meat is not important for them, they buy imported meat because of the quality or because they can buy high quantities, choose the more expensive grass-fed options for both loin and round, they had never heard of Novillo de Osorno, and they would not buy Novillo de Osorno.

The second group contained 299 respondents and included people of higher incomes (more than CLP 1 million), and most people who lived in La Araucanía, Los Lagos, or Los Ríos regions belonged to this cluster, that is, in the area in which Novillo de Osorno is reared. The frequency of older people (more than 50 years old) was lower than expected in this group. They are foodies, but they are not interested in meat. They love buying food and cooking and spend time on them. They use purchase lists and read the labels. They also love to eat out for pleasure, use new recipes, try new products, and like foreign recipes. Consequently, they do not think that traditional recipes are better than others. In addition, they are not interested in Chilean identity. Family meals are important for them and they do not eat out for work, but are not of fixed habits. They eat all kinds of meat and are the group more disposed to buy meat at the butchery inside a supermarket. Although price is not important for them, they choose the cheapest grass-fed option for the loin, maybe because taste is also unimportant but nutritive value is not. They had heard about Novillo de Osorno and they would buy it because it is free-range, although depending on the price. They could be named as Foodies uninvolved.

The third group was composed of 236 respondents and included people aged more than 55 years old, with low incomes (lower than CLP 750,000). People from the Northern (Arica, Coquimbo, Libertador, Maule, Tarapacá, and Valparaíso) regions are in this group. They could be named as Traditional since they like neither new recipes nor new products or foreign recipes. Consequently, they think that traditional recipes are better, that family meals are important, they do not like to eat out of the home, they have fixed habits, and they are interested in Chilean identity. They do not like to buy food, do not use a purchase list, do not trust in publicity, and do not read the labels. They eat all kinds of meat and buy it in trays at the supermarket, either national or imported. Since price and taste are more important for them than nutritive value, they choose the cheapest grass-fed option for both loin and round. They thought that Novillo de Osorno was dairy cattle, and although they do not buy it, they would buy Novillo de Osorno because of its prestige or origin or because they considered it as healthy.

#### 3.4.2. Clusters with Metropolitana de Santiago Region Data

Three classes were obtained from the hierarchical cluster. The clustering separated by gender, age, and income. Frequencies for the answers in each cluster are in Table A3.

Cluster 1 was composed of 94 respondents and included men of middle age (35–50 years old) and medium income (CLP 250,000–750,000). They are somewhat traditional and they like neither buying food nor cooking, spend little time on it, decide in the moment of purchase, and do not read the labels. It is the unique group that buys at the slaughterhouse, although they make purchases mainly from trays or in a specialized shop. They do not like new products, new recipes, or foreign recipes, think that traditional recipes are the best, and are interested in the Chilean identity of food. They are people of fixed habits, for whom meals with the family and friends are important. They do not like to eat out, whereas they must for work. Taste is more important for them than price or nutritive value. They eat mainly red meat, choose the cheapest grass-fed option for both the loin and the round, do not know the origin of the meat they buy, thought that Novillo de Osorno were dairy cattle, and they would buy Novillo de Osorno because of its prestige or origin. They could be named as Traditional men.

Cluster 2 contained 119 respondents, and was formed by females with incomes higher than CLP 1.5 million. They like to buy food and to cook and spend time on them. They read the labels, do not improvise in their purchases, like to try new products, are not interested in the Chilean identity of food, and do not think that traditional recipes are better. Neither families nor roasts are important for them, they have no fixed habits and they do not eat out for work. Taste is unimportant for them, but price and nutritional value are not. They buy meat in trays at the supermarket and choose the cheapest grass-fed options for loin and round, and did not know what Novillo de Osorno was but they would buy it depending on the price. We can name them as Females uninvolved.

Cluster 3 contained only 61 respondents and included young people (<35 years old), with high income (CLP 1–1.5 million). They like to buy food and cook, to eat out, and to try new recipes. They choose food by taste and roasts with friends are part of their social life, but they eat mainly white meats, which they buy in a specialized shop. They are the unique group that declare to eat meat of free-range animals. They choose the more expensive grass-fed loin and round, or the cheapest grain-fed options. They know that Novillo de Osorno is a kind of meat and did not express their purchase intention. They can be named Young uninvolved.

#### 3.4.3. Clusters with Regions Different from Metropolitana de Santiago Data

Three classes were obtained from the hierarchical cluster. Frequencies for the answers in each cluster are in Table A4. People were clustered by age and origin involvement.

The first group was composed of 113 respondents. They are people from 35 to 50 years old, with a high income (more than CLP 1 million), and who live mainly in the Aysén, La Araucanía, or Los Lagos regions, that is, the Southern area of the country, where Novillo de Osorno is reared. They like to buy food and to cook and spend time on them, make lists, and read the labels. Additionally, they like new products and recipes, including foreign recipes, and, consequently, they do not agree that traditional recipes are better, although they are concerned about the Chilean identity of food. Family is important for them. For them, nutritional value is more important than taste and they eat mainly white meats, which they purchase in traditional butcheries or from trays. They are also the unique group that buy meat at the slaughterhouse. They buy national meat and are interested in the origin of the meat they buy. They knew what Novillo de Osorno was through a friend or family comment and they buy Novillo de Osorno. In addition, with regard to purchase intention, taste and free-range rearing system are important. They could be named as Origin-concerned people.

The second group contained 136 respondents, and they were older people (more than 55 years old) with medium income (less than CLP 750,000), living in Arica, Atacama, Coquimbo, Maule, Tarapacá, and Valparaíso regions (north–middle area). They like neither cooking, nor eating out, and do not like new products or recipes or foreign recipes. They do not trust in publicity and do not read the labels. Family mealtime is important for them, they are people of fixed habits, they think that traditional recipes are the best, and they are concerned about the Chilean identity of food. Taste and price are more important for them than nutritional value. They eat all kinds of meat and buy it in trays, regardless of if national or imported, and buy imported meat because it is easier to find. They had never heard about Novillo de Osorno and they do not buy it, but they would buy Novillo de Osorno because of prestige, health, or origin. We will call them Traditional older.

The third group was formed by 92 respondents, young people (less than 35), with incomes from CLP 750,000 onwards. They live in the Biobío, La Araucanía, Los Ríos, and Magallanes regions (Southern). They like neither buying nor cooking food and do not spend time on them. They do not make purchase lists but trust in publicity and read the labels. They like to eat out and to try new products and recipes. Consequently, they do not think that traditional recipes are the best and they are not interested in the Chilean identity of food. They have no fixed habits and family mealtime is not important for them. They are not worried about the origin of the meat and buy either national or imported meat, the price being the criterion to buy imported meat. They declare to buy Novillo de Osorno sometimes and, in general, they have no inclination to buy it, the price being the unique criterion for which they would buy it. We will name them as Younger unconcerned.

From the three clusterings, we can see that young urban from clustering 1 are likely comparable to young uninvolved from clustering 2 and to young unconcerned from clustering 3. Additionally, foodies from clustering 1 are comparable to females from clustering 2. Finally, traditional from clustering 1 are similar to traditional from clustering 2 and to older traditional from clustering 3. Some patterns are repeated in all three analyses. Younger people trust advertising more than older people, they like to go out, and they do not have fixed habits. The young people of Santiago are distinguished from the other groups in that they like to shop and cook and in that price is less important than for young people in other regions. Foodies uninvolved and Females uninvolved have the highest income level, are not interested in Chilean identity, have no fixed habits, they like to buy food, to cook, and to try new products and recipes, they read the label, and they choose food for nutritional reasons. They differ in the degree of knowledge of Novillo de Osorno. Finally, traditional people seem to be men older than 55 with low income and living in the Northern areas. They like neither buying food nor cooking, they do not eat out for pleasure, do not trust in advertising, do not read the labels, and mealtime, traditional recipes, and Chilean identity are important for them. All of them would buy Novillo de Osorno for its prestige. Although, in general, the price is the criterion of choice, those from Santiago are also interested in taste.

When making food purchasing decisions, consumers consider both intrinsic and extrinsic attributes. Extrinsic attributes are not immediately detected and so, consumers must be informed about them through the label or through advertising [36]. Nevertheless, some studies showed that although only half of people state to read the labels, in fact, the actual use of the label is less than that declared. In addition, when people read the labels, the most searched-for pieces of information were calorie amount and nutritional information, such as fat or sugar content. Nevertheless, many people say they do not have knowledge enough about this nutritional information, which could discourage them from using the label information [37,38,39]. On the other hand, many people declare not relying on the information provided in the labelling, nor on the government entities responsible for their control [38]. There are gender differences in perceived food healthiness. Previous studies showed that women focus on the nutritional value of food [10] and prioritise healthy eating more than men [40,41].

Between the main reasons to choose a certain food are tradition and taste, as well as cultural elements [38,42], although price remains an important factor for certain segments of the population.

Concerning the Chilean identity and the knowledge of national products, previous findings with Chilean consumers showed that only 30% of respondents admitted to be concerned about where food is obtained [29]. Villalobos et al. [43] analysed the preferences of beef consumers in three areas of Chile and found that in Santiago, Chilean beef is less valued than Argentinian or Brazilian beef, whereas the opposite occurred in Rancagua and Talca. In addition, price was the criterion in Santiago and quality assurance in Rancagua and Talca. In other Chilean studies, it was established that consumers in the Metropolitan area of Santiago gave more importance to price than to origin, contrary to consumers in Araucanía [44], and price was detected as one of the main reasons to buy imported chicken meat [45]. Although several studies reported that women have stronger ethnocentric tendencies [46], a study developed in Chile reported no relationship between the degree of ethnocentrism and gender [47], whereas the older people have more ethnocentric tendencies than young [47], which agrees with the current results.

Many studies demonstrated the usefulness of clustering to find consumers’ behaviour patterns [7,35,48]. In a survey with Chilean consumers [47], the authors reported four clusters defined by city, frequency of purchase of imported foods, gender, area of residence, and ethnocentrism. Similarly, in another study by the same authors, they defined five groups of consumers with different social and food consumption habits and concluded that people showing a lifestyle with low involvement and enjoyment of food are associated with a higher socioeconomic status and lower age, which generally agrees with the current results [27].

## 4. Conclusions

Several practical implications can be drawn from the results.

Firstly, 57% of respondents had never heard about Novillo de Osorno and nearly 26% of the people that declared to know Novillo de Osorno were wrong about what it was. Therefore, the supply chain has an opportunity to extend the market, but it must be accompanied by a marketing strategy to inform consumers of what Novillo de Osorno really is. Since price and confidence in the origin are important cues, they must be considered in the design of these promotion strategies.

Secondly, the Chilean supply chain has the challenge to increase internal sales, since part of the population declare that they do not find Chilean meat at the markets. Of the respondents, 20% thought that foreign meat has a better quality than Chilean meat, which implies that the suppliers should make an effort to transmit to the population the actual quality of Chilean meat. Several private and institutional strategies could be implemented for this purpose.

On the other hand, the structure of the families (number of adults and children at home) was less significant than residence place and consumer gender, age, or income in defining consumers’ lifestyles, meat consumption, and purchase habits. 

People can be grouped by their socio-demographic and lifestyle characteristics, that should be taken into account when designing marketing strategies: 1. Younger people: urban with medium-high incomes, which search only for pleasure; 2. Foodies uninvolved and Females uninvolved: females with the highest income level that choose food for nutritional reasons; and 3. Traditional people: men older than 55 with low incomes, living in the Northern areas and interested in taste and in the meat’s origin.

## Figures and Tables

**Table 1:** The percentages of population by administrative region in the whole country and in the current sample.

Country Region	Country	Sample
Antofagasta	3.5	2.1
Arica y Parinacota	1.3	1.0
Atacama	1.6	1.6
Aysén del General Carlos Ibáñez Del Campo	0.6	1.1
Biobío	8.9	11.6
Coquimbo	4.3	2.8
La Araucanía	5.4	6.0
Libertador General Bernardo O’Higgins	5.2	4.7
Los Lagos	4.7	9.3
Los Ríos	2.2	2.3
Magallanes y de La Antártica Chilena	0.9	1.8
Maule	5.9	4.1
Metropolitana de Santiago	40.5	44.6
Tarapacá	1.9	1.8
Valparaíso	10.3	7.3

**Table 2:** The *p* values for the chi-square in the Kruskal–Wallis test with the socio-demographic variables (number of children/adults at home and consumer gender, age, and income) as main effects.

	Adults	Children	Gender	Age	Income
FOOD LIFESTYLE					
I like to buy food	0.243	0.428	**0.005**	0.117	0.302
I trust more in a product if I have seen it in advertising	0.143	0.185	0.245	**0.033**	**0.015**
I do not spend too much time on food purchases	0.738	0.777	**0.012**	0.034	0.076
I compare the information from the labels	0.167	0.308	0.115	**0.015**	**<0.001**
Since I have no health problems, the taste is the most important for me	0.171	0.129	**0.048**	0.377	**<0.001**
For me, price is the most important	0.435	0.443	0.450	0.086	**<0.001**
I always make a list before I go to buy food	0.101	0.296	0.606	0.064	0.096
I decide what to buy when I am already in the store, depending on what I see	0.835	0.162	0.403	0.059	0.085
I am interested in the recovery of regional ingredients with Chilean identity	0.207	0.234	0.057	**0.033**	**<0.001**
I like to try products that I have never bought	0.895	0.958	0.484	**<0.001**	**<0.001**
I choose the products for their nutritional value more than by their taste	0.413	0.397	**<0.001**	0.203	**0.003**
We frequently eat out of the house, we love it	0.377	0.620	0.723	**<0.001**	**<0.001**
Mealtime is very important in my house because it brings together the family	0.268	**0.045**	0.378	**<0.001**	**<0.001**
I love cooking	0.271	0.435	**0.030**	0.593	0.018
The best recipes are traditional ones	**0.003**	0.251	0.524	0.084	**0.000**
Roasts with friends are an important part of my social life	0.923	0.724	**0.001**	**<0.001**	**0.009**
I am a person of fixed habits at mealtime	0.121	0.272	0.921	**<0.001**	**<0.001**
I eat out because work demands it, but I would rather eat at home	**0.050**	0.837	0.129	**0.002**	**<0.001**
I like to try new recipes	0.499	0.897	0.473	**<0.001**	**<0.001**
I do not spend time cooking; I prefer to do other things	**0.005**	0.072	**0.005**	0.437	**0.007**
I like to cook other countries’ or cultures’ recipes	0.257	0.267	0.160	**0.016**	0.193
MEAT CONSUMPTION AND PURCHASE HABITS					
What kind of meat do you eat?	0.950	0.624	0.051	0.451	**0.001**
Where do you usually buy the meat?	0.878	**0.011**	0.308	0.320	0.509
Do you know the origin of the meat?	0.531	0.154	0.232	**0.002**	**0.008**
Why do you buy imported meat?	0.879	0.370	0.355	0.201	0.711
MEAT CHOICE BEHAVIOUR					
Loin choice	0.472	0.716	0.958	0.140	0.141
Round choice	**0.037**	0.612	0.064	**0.025**	0.651
NOVILLO DE OSORNO KNOWLEDGE AND WILLINGNESS TO PURCHASE IT					
Have you heard of Novillo de Osorno? *	0.106	0.149	**0.010**	**<0.001**	**0.001**
What do you think Novillo de Osorno is?	0.475	0.270	0.599	**0.005**	0.077
Would the meat you buy be considered as Novillo de Osorno?	0.194	0.574	0.539	0.623	0.485
Would you buy it? *	0.959	0.443	0.984	**0.043**	0.094

* For the analysis, this question was recorded as a binary variable (yes/no). *p*-values < 0.05 are marked in bold.

## Appendix A

**Table A1:** Questionnaire used in the survey and the valid percentage of answers. In true/false questions, only the true option percentage is shown.

FOOD LIFESTYLE	*% True*
**Making the purchase, each of us have our own organization and value different things about the product. Of the options below, tell us which ones describe how you buy**. *(True/false)*	
I like to buy food	76.1
I trust more in a product if I have seen it in advertising	20.3
I do not spend too much time on food purchases	57.7
I compare the information from the labels	59.3
Since I have no health problems, the taste is the most important for me	61.8
For me, price is the most important	41.6
I always make a list before I go to buy food	54.3
I decide what to buy when I am already in the store, depending on what I see	64.4
I am interested in the recovery of regional ingredients with Chilean identity	83.6
I like to try products that I have never bought	71.2
I choose the products for their nutritional value more than by their taste	43.1
**In each house, the eating habits are different. With which of the following statements do you most identify?** (*True/false)*	
We frequently eat out of house, we love it	22.6
Mealtime is very important in my house because it brings together the family	81.1
I love cooking	61.8
The best recipes are traditional ones	76.6
Roasts with friends are an important part of my social life	66.2
I am a person of fixed habits at mealtime	60
I eat out because work demands it, but I would rather eat at home	70.7
I like to try new recipes	78.2
I do not spend time cooking, I prefer to do other things	30.1
I like to cook other countries’ or cultures’ recipes	53.2
MEAT CONSUMPTION AND PURCHASE HABITS	*valid %*
**What kind of meat do you eat?** (*Mark only one oval)*	
Mainly, white meat (chicken, pork, turkey...)	12.6
Mainly, white meat (chicken, pork, turkey...) but only from free-range animals	1.3
Mainly, red meat (beef, lamb…)	10.2
Mainly, red meat (beef, lamb…) but only from free-range animals	1.6
All kind of meats	74.1
All kind of meats but only from free-range animals	1.8
**Where do you usually buy the meat?** (*Mark only one oval)*	
I have my own animals	1.7
Directly from the farmer	0.7
In a slaughterhouse	1.2
At the supermarket, on trays	44.8
In the butchery inside a supermarket	14.6
In a shop specializing in meat	8.2
In a traditional butchery	28.7
**Do you know the origin of the meat you buy; where it comes from?** *(**Mark only one oval)*	
I only buy meat from the region where I live	8.4
I only buy meat of national origin, from any region	21.3
I only buy imported meat	4.5
Sometimes it is national meat and sometimes imported	48.8
No, because it is hard to know where it is coming from	6.7
I never ask; for me, the origin is not important	10.4
**Why do you buy imported meat?** *(**Mark only one oval)*	
It is cheaper	33.7
It has better quality	18.8
It is the one I find most easily	32.2
I trust more in the health of the animals	1.0
Because it has a constant price, without large fluctuations	5.4
Because I can buy large quantities	1.0
Because I can buy it frozen	7.9
Because it is fashionable	0.0
MEAT CHOICE BEHAVIOUR	
**Imagine you are in the shop and find the following types of loin. Which one would you choose?** *(Mark only one oval)*	

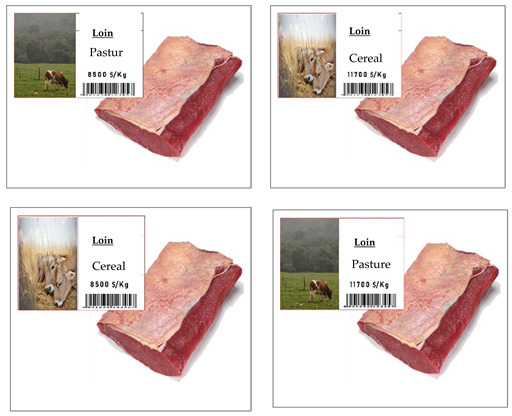

**Imagine you are in the shop and find the following types of round. Which one would you choose?** (*Mark only one oval*)	

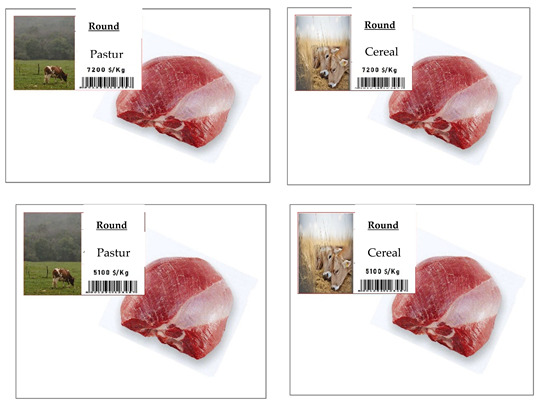

NOVILLO DE OSORNO KNOWLEDGE AND WILLINGNESS TO PURCHASE IT	*valid %*
**Have you heard of Novillo de Osorno?** *(**Mark only one oval)*	
Yes, by the paper, radio, or on television	9.7
Yes, by the Internet or social media	6.2
Yes, a family member or friend commented on something	7.4
Yes, I know the product	18.3
Yes, by the butcher or restaurant	1.7
I have never heard of it	56.7
**What do you think Novillo de Osorno is?** *(* *Mark only one oval)*	
Dairy cattle	26.4
A kind of meat	36.1
A breed	16.5
The name of a field dedicated to livestock	6.5
I am not sure	14.5
**Novillo de Osorno are animals raised between Lanco * and Los Muermos * and fed on grassland** *(**informative, no question)**(** *Lanco and Los Muermos are two cities of the Novillo de Osorno breeding area)*	
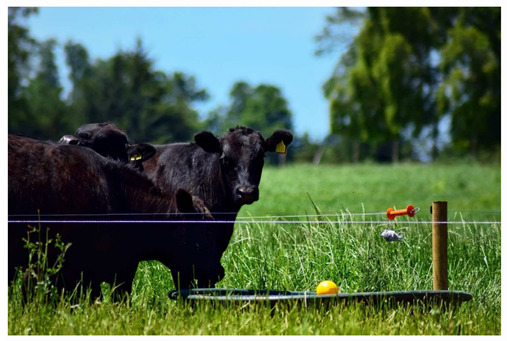
**Following this definition, would the meat you buy be considered as Novillo de Osorno?** *(**Mark only one oval)*	
Yes	14.8
No	38.0
Sometimes	19.2
I do not know, because I do not know where the meat I buy comes from	28.0
**Imagine that in your usual shop there was Novillo de Osorno. Would you buy it?** *(Mark only one oval)*	
Yes, for its taste	6.0
Yes, for its prestige	11.4
Yes, because I trust in its origin	32.4
Yes, because it is healthier	14.1
Yes, because it comes from free-range animals	8.0
Sometimes, depending on the piece and price	25.5
No, because the meat is all the same	2.6
SOCIO-DEMOGRAPHIC VARIABLES	
**In which city do you live? ^1^** *(open answer)*	
Antofagasta	2.1
Arica y Parinacota	1.0
Atacama	1.6
Aysén del General Carlos Ibáñez Del Campo	1.1
Biobío	11.6
Coquimbo	2.8
La Araucanía	6.0
Libertador General Bernardo O’Higgins	4.7
Los Lagos	9.3
Los Ríos	2.3
Magallanes y de La Antártica Chilena	1.8
Maule	4.1
Metropolitana de Santiago	44.6
Tarapacá	1.8
Valparaíso	7.3
**Age** ^2^*(open answer, only two-digit numbers are possible)*	
18–34	33.3
35–50	36.3
51–83	30.4
**Gender** (*men/women)*	
Men	49.8
Women	50.2
**How many minors live in your home (0–18 years)?** ^3^ *(open answer, only numbers are possible)*	
0	57.9
1	24.9
2	11.5
3	4.9
4	0.7
5	0.0
6	0.2
**How many adults live in your home, including you?** ^3^*(open answer, only numbers are possible)*	
1	12.3
2	43.4
3	20.3
4	16.1
5	5.2
6	2.1
7	0.5
**Could you tell us your family group’s monthly rent?** ^4^ *(**Mark only one oval)*	
No income	0.7
Less than CLP 250,000	2.9
CLP 250,000–500,000	19.4
CLP 500,000–750,000	19.4
CLP 750,000–1 million	12.4
CLP 1–1.5 million	12.7
More than CLP 1.5 million	32.5

^1^ Residence places were recorded considering the regions of belonging, considering the administrative divisions of the country. ^2^ Consumer age was recorded in three categories, accordingly with national statistics of population distribution. ^3^ Open answer. We show here the percentages for valid answers. ^4^ CLP 1000 is approximately USD 1.26. The average monthly income in Chile was CLP 573,964 in 2018. The median income reached CLP 400,000 per month (National Statistics Institute).

**Table A2:** The frequencies of answers for each item in each cluster when all data were used. Only items for which differences between clusters’ frequencies were significant are shown.

	Young Urban (*n* = 80)	Foodies (*n* = 299)	Traditional (*n* = 236)	*p*
I like to buy food *	*65.0*	**83.3**	*70.8*	<0.001
I trust more in a product if I have seen it in advertising	**33.8**	21.1	*14.8*	0.001
I do not spend too much time on food purchases	**81.3**	*48.2*	61.9	<0.001
I compare the information from the labels	55.0	**82.3**	*31.8*	<0.001
Since I have no health problems, the taste is the most important for me	**76.3**	*41.5*	**82.6**	<0.001
For me, price is the most important	37.5	*23.4*	**66.1**	<0.001
I always make a list before I go to buy food	*35.0*	**61.9**	51.3	<0.001
I decide what to buy when I am already in the store, depending on what I see	**77.5**	*55.2*	**71.6**	<0.001
I am interested in the recovery of regional ingredients with Chilean identity	*67.5*	*77.9*	**96.2**	<0.001
I like to try products that I have never bought	73.8	**83.3**	*55.1*	<0.001
I choose the products for their nutritional value more than by their taste	46.3	**57.9**	*23.3*	<0.001
We frequently eat out of house, we love it	**57.5**	**26.4**	*5.9*	<0.001
Mealtime is very important in my house because it brings together the family	*56.3*	**75.6**	**96.6**	<0.001
I love cooking	*38.8*	**69.9**	59.3	<0.001
The best recipes are traditional ones	75.0	60.5	**97.5**	<0.001
I am a person of fixed habits at mealtime	*40.0*	*45.2*	**85.6**	<0.001
I eat out because work demands it, but I would rather eat at home	**87.5**	*67.6*	69.1	0.002
I like to try new recipes	77.5	**90.3**	*63.1*	<0.001
I do not spend time cooking; I prefer to do another thing	**62.5**	*15.7*	**37.3**	<0.001
I like to cook other countries’ or cultures’ recipes	*36.3*	**65.2**	*43.6*	<0.001
**Consumed meat**				
Mainly, white meat (chicken, pork, turkey...)	**22.5**	12.4	*5.1*	<0.001
Mainly, white meat (chicken, pork, turkey...) but only from free-range animals	**3.8**	1.7	*0.0*	<0.001
Mainly, red meat (beef, lamb…)	15.0	10.0	8.9	<0.001
Mainly, red meat (beef, lamb…) but only from free-range animals	3.8	2.0	0.4	<0.001
All kind of meats	*52.5*	**71.2**	**85.2**	<0.001
All kind of meats but only from free-range animals	2.5	2.7	*0.4*	<0.001
**Purchase place**				
I have my own animals	*0.0*	1.1	**6.5**	<0.001
Directly from the farmer	*0.0*	1.1	*0.0*	<0.001
In a slaughterhouse	2.5	1.1	*0.0*	<0.001
At the supermarket, on trays	*32.5*	43.9	**64.5**	<0.001
In the butchery inside a supermarket	11.3	**17.6**	*6.5*	<0.001
In a shop specializing in meat	7.5	9.2	4.8	<0.001
In a traditional butchery	**46.3**	26.0	*17.7*	<0.001
**Origin of the meat**				
I only buy meat from the region where I live	*2.5*	9.9	9.7	<0.001
I only buy meat of national origin, from any region	20.0	**25.2**	*6.5*	<0.001
I only buy imported meat	**12.5**	*2.7*	1.6	<0.001
Sometimes it is national meat and sometimes imported	*28.8*	51.1	**64.5**	<0.001
No, because it is hard to know where it is coming from	3.8	5.0	**17.7**	<0.001
I never ask; for me, the origin is not important	**32.5**	*6.1*	*0.0*	<0.001
**Pictures choice**				
Loin 1	48.8	**79.8**	**85.5**	<0.001
Loin 2	**40.0**	*13.4*	*1.6*	
Loin 3	8.8	5.3	**12.9**	
Loin 4	2.5	1.5	*0.0*	
Round 1	*46.3*	71.0	**87.1**	<0.001
Round 2	**45.0**	21.4	4.8	
Round 3	8.8	6.5	4.8	
Round 4	*0.0*	1.1	3.2	
**Have you heard of Novillo de Osorno?**				<0.001
Yes, by the paper, radio, or on television	*2.5*	11.5	11.3	
Yes, by the Internet or social media	3.8	7.3	4.8	
Yes, a family member or friend commented on something	8.8	7.6	4.8	
Yes, I know the product	*8.8*	20.2	22.6	
Yes, by the butcher or restaurant	2.5	1.5	1.6	
I have never heard of it	**73.8**	*51.9*	54.8	
**Novillo de Osorno is…**				0.003
Dairy cattle	*5.6*	*14.8*	**36.2**	
A kind of meat	**66.7**	**43.7**	*28.1*	
A breed	11.1	12.6	19.6	
The name of a field dedicated to livestock	11.1	4.4	7.5	
I am not sure	5.6	**24.4**	*8.5*	
**Do you buy Novillo de Osorno?**				<0.001
Yes	10.0	**21.1**	*8.5*	
No	41.3	*28.4*	**49.2**	
Sometimes	18.8	**29.1**	*6.8*	
I do not know	30.0	*21.4*	**35.6**	
**Would you buy Novillo de Osorno?**				<0.001
Yes, for its taste	**11.3**	5.4	5.1	
Yes, for its prestige	*3.8*	*5.0*	**22.0**	
Yes, because it is healthier	8.8	*10.4*	**20.8**	
Yes, because I trust in its origin	25.0	**27.4**	**41.1**	
Yes, because it comes from free-range animals	11.3	**11.7**	*2.1*	
Sometimes, depending on the piece and price	33.8	**37.5**	*7.6*	
No, because the meat is all the same	**6.3**	2.7	1.3	
**Region**				<0.001
Antofagasta	*0.0*	1.7	3.4	
Arica y Parinacota	*0.0*	0.3	**2.1**	
Atacama	*0.0*	0.3	**3.8**	
Aysén del General Carlos Ibáñez Del Campo	2.5	1.7	*0.0*	
Biobío	12.5	8.7	9.7	
Coquimbo	*0.0*	*0.3*	6.8	
La Araucanía	6.3	**9.7**	1.3	
Libertador General Bernardo O’Higgins	1.3	**3.0**	8.1	
Los Lagos	*1.3*	**15.1**	*4.7*	
Los Ríos	2.5	**3.7**	0.4	
Magallanes y de La Antártica Chilena	1.3	2.7	0.8	
Maule	*0.0*	3.3	**6.4**	
Metropolitana de Santiago	**72.5**	46.2	**33.1**	
Tarapacá	*0.0*	1.0	**3.4**	
Valparaíso	*0.0*	2.3	**16.1**	
**Age**				
18–34	**60.0**	37.1	*19.5*	<0.001
35–50	*20.0*	38.8	38.6	
51–83	*20.0*	*24.1*	**41.9**	
**Income**				
No income	**2.5**	0.7	5.1	<0.001
Less than CLP 250,000 *	1.3	1.7	**41.5**	
CLP 250,000–500,000	*5.0*	5.7	**30.5**	
CLP 500,000–750,000	15.0	*11.8*	**8.9**	
CLP 750,000–1 million	**22.5**	12.5	*3.8*	
CLP 1–1.5 million	17.5	**18.5**	*10.2*	
More than CLP 1.5 million	36.3	**49.2**	*5.1*	

* In true/false items, only the percentage of true responses is shown. Frequencies higher than expected are in bold. Frequencies lower than expected are in italic.

**Table A3:** The frequencies of answers for each item in each cluster when only data from the Metropolitana de Santiago region were used. Only items for which differences between clusters’ frequencies were significant are shown.

	Traditional Men (*n* = 94)	Females Uninvolved (*n* = 119)	Young Uninvolved (*n* = 61)	*p*
I like to buy food	*61.7*	**82.4**	**86.9**	<0.001
I do not spend too much time on food purchases	**85.1**	*52.1*	67.2	<0.001
I compare the information from the labels	*21.3*	**84.9**	67.2	<0.001
Since I have no health problems, the taste is the most important for me	**78.7**	*32.8*	**86.9**	<0.001
For me, price is the most important	*47.9*	**30.3**	39.3	0.032
I always make a list before I go to buy food	44.7	61.3	55.7	0.052
I decide what to buy when I am already in the store, depending on what I see	**75.5**	*58.0*	68.9	0.024
I am interested in the recovery of regional ingredients with Chilean identity	**89.4**	*63.0*	85.2	<0.001
I like to try products that I have never bought	*62.8*	**84.0**	85.2	<0.001
I choose the products for their nutritional value more than by their taste	*38.3*	**60.5**	55.7	0.004
We frequently eat out of house, we love it	*21.3*	33.6	**45.9**	0.005
Mealtime is very important in my house because it brings together the family	**90.4**	*63.0*	78.7	<0.001
I love cooking	*38.3*	**67.2**	**78.7**	<0.001
The best recipes are traditional ones	**97.9**	*54.6*	82.0	<0.001
Roasts with friends are an important part of my social life	**69.1**	*39.5*	**67.2**	<0.001
I am a person of fixed habits at mealtime	**74.5**	*48.7*	*41.0*	<0.001
I eat out because work demands it, but I would rather eat at home	**88.3**	*67.2*	*96.7*	<0.001
I like to try new recipes	*76.6*	85.7	**93.4**	0.016
I do not spend time cooking; I prefer to do other things	**55.3**	*21.8*	31.1	<0.001
I like to cook other countries’ or cultures’ recipes	*43.6*	**68.1**	65.6	0.001
**Consumed meat**				
Mainly, white meat (chicken, pork, turkey...)	*7.4*	15.1	**29.5**	0.001
Mainly, white meat (chicken, pork, turkey...) but only from free-range animals	1.1	5.0	1.6	
Mainly, red meat (beef, lamb…)	9.6	5.9	**16.4**	
Mainly, red meat (beef, lamb…) but only from free-range animals		1.7	3.3	
All kind of meats	**81.9**	70.6	*42.6*	
All kind of meats but only from free-range animals	0.0	1.7	**6.6**	
**Purchase place**				0.002
I have my own animals	0.0	0.0	0.0	
Directly from the farmer	0.0	1.0	1.6	
In a slaughterhouse	**2.8**	0.0	0.0	
At the supermarket, on trays	47.2	**58.3**	*32.8*	
In the butchery inside a supermarket	8.3	**22.9**	14.8	
In a shop specializing in meat	38.9	*12.5*	**47.5**	
In a traditional butchery	2.8	5.2	3.3	
**Picture choice**				<0.001
Loin 1	**83.3**	**84.4**	*31.1*	
Loin 2	13.9	*12.5*	**47.5**	
Loin 3	2.8	*2.1*	**18.0**	
Loin 4	0.0	1.0	3.3	
Round 1	**83.3**	**80.2**	*21.3*	
Round 2	*16.7*	*16.7*	**63.9**	
Round 3	0.0	*2.1*	**13.1**	
Round 4	0.0	1.0	1.6	
**Novillo de Osorno is…**				0.003
Dairy cattle	**40.6**	*15.2*	*0.0*	
A kind of meat	21.7	28.3	**63.6**	
A breed	20.3	10.9	9.1	
The name of a field dedicated to livestock	5.8	10.9	18.2	
I am not sure	11.6	**34.8**	9.1	
**Would you buy Novillo de Osorno?**				<0.001
Yes, for its taste	6.4	3.4	11.5	
Yes, for its prestige	**17.0**	*5.0*	3.3	
Yes, because I trust in its origin	**37.2**	*21.8*	31.1	
Yes, because it is healthier	19.1	*8.4*	16.4	
Yes, because it comes from free-range animals	*5.3*	13.4	13.1	
Sometimes, depending on the piece and price	*10.6*	**42.0**	21.3	
No, because the meat is all the same	4.3	5.9	3.3	
**Age**				
18–34	*33.0*	46.2	**57.4**	0.018
35–50	**35.1**	21.8	24.6	
51–83	31.9	31.9	*18.0*	
**Gender**				
Men	**59.6**	41.2	50.8	0.028
Women	40.4	**58.8**	49.2	
**Income**				
No income	2.1	0.8	0.0	<0.001
Less than CLP 250,000	2.1	5.9	1.6	
CLP 250,000–500,000	**22.3**	*9.3*	9.8	
CLP 500,000–750,000	**41.5**	*12.7*	23.0	
CLP 750,000–1 million	12.8	16.1	19.7	
CLP 1–1.5 million	*4.3*	**12.7**	**18.0**	
More than CLP 1.5 million	*14.9*	**42.4**	27.9	

Frequencies higher than expected are in bold. Frequencies lower than expected are in italic.

**Table A4:** The frequencies of answers for each item in each cluster when data from the regions different from Metropolitana de Santiago were used. Only items for which differences between clusters’ frequencies were significant are shown.

	Origin Concerned (*n* = 113)	Traditional Older (*n* = 136)	Young Unconcerned (*n* = 92)	*p*
I like to buy food	**86.7**	74.3	*65.2*	0.001
I trust more in a product if I have seen it in advertising	22.1	*13.2*	**32.6**	<0.001
I do not spend too much time on food purchases	43.4	50.7	**58.7**	<0.001
I compare the information from the labels	**89.4**	*22.1*	**78.3**	<0.001
Since I have no health problems, the taste is the most important for me	*32.7*	**91.9**	56.5	<0.001
For me, price is the most important	*24.8*	**76.5**	*20.7*	<0.001
I always make a list before I go to buy food	**75.2**	50.0	*34.8*	<0.001
I decide what to buy when I am already in the store, depending on what I see	*51.3*	67.6	69.6	<0.001
I am interested in the recovery of regional ingredients with Chilean identity	**96.5**	**97.1**	*67.4*	<0.001
I like to try products that I have never bought	**77.0**	*51.5*	**76.1**	<0.001
I choose the products for their nutritional value more than by their taste	**63.7**	*10.3*	40.1	<0.001
We frequently eat out of house, we love it	14.2	*5.9*	**29.3**	<0.001
Mealtime is very important in my house because it brings together the family	**94.7**	**94.9**	*59.8*	<0.001
I love cooking	**72.6**	66.9	*46.7*	<0.001
The best recipes are traditional ones	*69.9*	**97.8**	*56.5*	<0.001
I am a person of fixed habits at mealtime	57.5	**84.6**	*39.1*	<0.001
I like to try new recipes	**85.0**	*58.1*	**81.5**	<0.001
I do not spend time cooking; I prefer to do other things	*10.6*	**38.2**	26.1	<0.001
I like to cook other countries’ or cultures’ recipes	**59.3**	*40.4*	46.7	0.012
Consumed meat				
Mainly, white meat (chicken, pork, turkey...)	**13.3**	*1.5*	7.6	<0.001
Mainly, white meat (chicken, pork, turkey...) but only from free-range animals	0.0	0.0	0.0	
Mainly, red meat (beef, lamb…)	10.6	8.1	15.2	
Mainly, red meat (beef, lamb…) but only from free-range animals	3.5	0.7	1.1	
All kind of meats	*69.9*	**88.2**	76.1	
All kind of meats but only from free-range animals	2.7	1.5	0.0	
Purchase place				
I have my own animals	2.9	**14.8**	*0.0*	0.040
Directly from the farmer	0.0	0.0	1.2	
In a slaughterhouse	**3.9**	0.0	0.0	
At the supermarket, on trays	36.3	44.4	47.6	
In the butchery inside a supermarket	8.8	18.5	13.4	
In a shop specializing in meat	16.7	0.0	9.8	
In a traditional butchery	**31.4**	*22.2*	28.0	
Origin of the bought meat				
I only buy meat from the region where I live	**25.5**	14.8	1.2	<0.001
I only buy meat of national origin, from any region	**37.3**	*0.0*	23.2	
I only buy imported meat	0.0	0.0	2.4	
Sometimes it is national meat and sometimes imported	*30.4*	**66.7**	**59.8**	
No, because it is hard to know where it is coming from	5.9	*18.5*	4.9	
I never ask; for me, the origin is not important	*1.0*	0.0	**8.5**	
Reason for importing meat				
It is cheaper	*20.7*	27.8	**53.1**	0.023
It has better quality	6.9	0.0	6.1	
It is the one I find most easily	48.3	**55.6**	26.5	
I trust more in the health of the animals	0.0	5.6	0.0	
Because it has a constant price, without large fluctuations	3.4	5.6	*4.1*	
Because I can buy large quantities	3.4	0.0	0.0	
Because I can buy it frozen	17.2	5.6	10.2	
Have you heard of Novillo de Osorno?				
Yes, by the paper, radio, or on television	12.7	14.8	13.4	0.028
Yes, by the Internet or social media	8.8	0.0	6.1	
Yes, a family member or friend commented on something	**14.7**	3.7	7.3	
Yes, I know the product	*30.4*	14.8	19.5	
Yes, by the butcher or restaurant	0.0	0.0	3.7	
I have never heard of it	*33.3*	**66.7**	50.0	
Do you buy Novillo de Osorno?				
Yes	**33.6**	*5.9*	18.5	0.006
No	*20.4*	**57.4**	*22.8*	
Sometimes	**31.9**	*5.1*	**27.2**	
I do not know	*14.2*	**31.6**	31.5	
Would you buy Novillo de Osorno?				
Yes, for its taste	**10.6**	*2.9*	4.3	<0.001
Yes, for its prestige	*8.0*	**23.5**	*5.4*	
Yes, because I trust in its origin	31.9	**45.6**	*22.8*	
Yes, because it is healthier	14.2	**19.1**	*7.6*	
Yes, because it comes from free-range animals	**13.3**	*2.2*	2.2	
Sometimes, depending on the piece and price	22.1	*6.6*	**54.3**	
No, because the meat is all the same	0.0	0.0	**3.3**	
Region				
Antofagasta	2.7	4.4	4.3	0.052
Arica y Parinacota	0.0	**3.7**	1.1	
Atacama	0.9	**5.9**	1.1	
Aysén del General Carlos Ibáñez Del Campo	**6.2**	*0.0*	0.0	
Biobío	13.3	14.7	**26.1**	
Coquimbo	*1.8*	**11.0**	*0.0*	
La Araucanía	**18.6**	*0.0*	**17.4**	
Libertador General Bernardo O’Higgins	7.1	11.0	6.5	
Los Lagos	**28.3**	*4.4*	20.7	
Los Ríos	5.3	*0.7*	**7.6**	
Magallanes y de La Antártica Chilena	2.7	*0.7*	**7.6**	
Maule	6.2	**11.0**	*3.3*	
Tarapacá	1.8	**6.6**	*0.0*	
Valparaíso	*5.3*	**25.7**	*4.3*	
Age				
18–34	24.8	*18.4*	**33.7**	0.002
35–50	49.6	39.7	42.4	
51–83	25.7	**41.9**	*23.9*	
Income				
No income	0.9	0.0	0.0	<0.001
Less than CLP 250,000	0.0	**5.9**	0.0	
CLP 250,000–500,000	*5.4*	**52.9**	*3.3*	
CLP 500,000–750,000	*8.0*	**26.5**	*6.5*	
CLP 750,000–1 million	10.7	*4.4*	**16.3**	
CLP 1 million–1.5 million	**20.5**	*4.4*	**20.7**	
More than CLP 1.5 million	**54.5**	*5.9*	**53.3**	

Frequencies higher than expected are in bold. Frequencies lower than expected are in italic.
